# Left Atrial Appendage Dysfunction in a Patient with Premature Ventricular Contractions - A Risk Factor for Stroke?

**DOI:** 10.1016/s0972-6292(16)30647-7

**Published:** 2013-08-01

**Authors:** Sandeep M Patel, Michael J Ackerman, Samuel J Asirvatham

**Affiliations:** 1 Department of Internal Medicine, Mayo Clinic, Rochester, Minnesota; 2 Division of Cardiovascular Diseases, Mayo Clinic, Rochester, Minnesota; 3 Department of Pediatrics and Adolescent Medicine/Division of Pediatric Cardiology, Mayo Clinic, Rochester, Minnesota

**Keywords:** PVC, left atrial appendage, stroke, risk, noncompaction cardiomyopathy

## Abstract

A 16-year-old female with ventricular dysfunction and frequent ventricular arrhythmia presented with a cardioembolic stroke. Prior electrophysiology study and ablation was performed for ventricular tachycardia (VT). For remaining ventricular ectopy, the patient was maintained on carvedilol and mexiletine. After one year on this regimen, she presented with an acute stroke. Transesophageal echocardiography revealed no evidence of an intracardiac or ventricular thrombus but demonstrated markedly decreased left atrial appendage (LAA) flow velocity worsened during frequent premature ventricular contractions (PVC). In the absence of atrial fibrillation (AF), the LAA dysfunction was considered secondary to the frequent PVCs and was thought to be the underlying cause for the stroke. We present this case to highlight a potential under recognized association between LAA dysfunction and ventricular arrhythmia, similar to that observed with atrioventricular dyssynchronous pacing.

## Introduction

LAA dysfunction in the setting of AF is a well-established risk factor for stroke [1]. Echocardiographically determined slowing of LAA emptying flow velocity likely leads to stasis, thrombus formation, and subsequent embolization. Similarly, patients with asynchronous cardiac pacing (VVI) have atrial morphological and electrical remodeling that results in LAA dysfunction [2]. Whether or not PVCs that also produce atrioventricular dyssynchrony have a similar association with thromboembolic stroke as with long-term VVI pacing is unknown. We present a patient with ventricular dysfunction and frequent PVCs, who presented with an acute stroke one year after radiofrequency ablation for VT. The evaluation was significant for severely decreased LAA emptying velocity, particularly during PVCs. We discuss the relevant literature and the possibility that frequent PVCs may be an under recognized substrate for LAA dysfunction with increased propensity for stroke.

## Case Presentation

**Figure 1:**
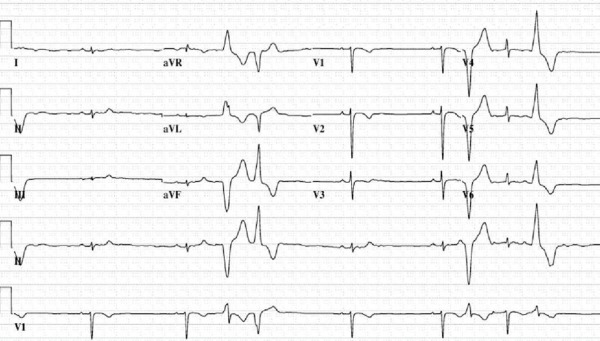
Electrocardiogram demonstrating a pair of PVCs with different morphologies and two isolated PVCs indicating multifocal etiology.

A 16-year-old female presented with exertional dyspnea and presyncope. Frequent PVCs were noted on exam and electrocardiogram (ECG, Figure 1). Different morphologies consistent with left ventricular (LV) origin were found. Initial transthoracic echocardiography demonstrated ejection fraction of 52% with mild right ventricular (RV) enlargement, and hypokinesis of the mid-to-distal RV free wall. MRI imaging showed no evidence of fibro-fatty infiltration of the RV or other evidence of arrhythmogenic right ventricular cardiomyopathy. A 24-hour Holter monitoring showed 71, 290 PVCs (41% of total beats) and non-sustained VT (8 beats, 170 beats per minute). With exercise testing, ventricular ectopy increased in frequency was multi-focal, and several 3-5 beat runs of nonsustained VT were noted. Because of this, a diagnostic electrophysiologic (EP) study for risk assessment and to determine potential need for ablation, antiarrhythmic therapy, or automated internal cardiac defibrillator (AICD) implantation was performed. At EP study, sustained monomorphic VT of right bundle superior axis was repeatedly induced and targeted successfully for ablation. Multiple PVCs were noted and were not mapped or ablated. AICD was considered but based on patient preference and the lack of inducible VT following ablation, an AICD was not placed.

Six months later, the patient continued to have frequent ectopy (14, 748 beats/24 hours) without sustained VT but now with decreased ventricular function (40% ejection fraction). The possibility of PVC-related cardiomyopathy was considered and a repeat EP study was performed. Sustained VT was no longer inducible, and two of several PVC origins were targeted and ablated successfully. Lidocaine was used at EP study, and PVCs were noted to decrease. Therefore, the patient was maintained on a medical regimen that included carvedilol and mexiletine. Warfarin was used for three months post LV ablation and then discontinued.

**Figure 2:**
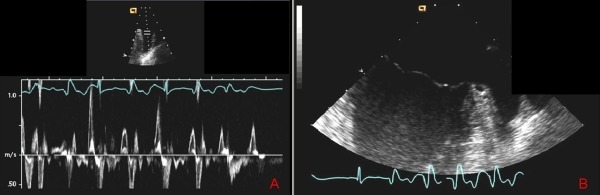
Transesophageal echocardiogram with LAA flow velocities (A) and multi-lobulated LAA (B) showing an almost 50% decrease in flow velocity during the PVCs and normal velocity during sinus rhythm (third and fifth beats) without evidence of intra-cardiac thrombus or spontaneous echo-contrast.

The patient did well on this regimen for one year when she presented to her local emergency department with new left-sided weakness, dysarthria, and facial droop. CT and subsequent MRI with diffusion-weighted imaging demonstrated infarction of the posterior right globus pallidus and putamen that extended into the posterior limb of the internal capsule. The visualization of multiple areas of infarct strongly suggested a cardioembolic source. Transesophageal echocardiography demonstrated a dilated LAA with markedly reduced LAA flow velocities, particular during frequent ventricular ectopic beats (Figure 2). She was treated for embolic stroke and dismissed on warfarin and rehabilitation.

**Figure 3:**
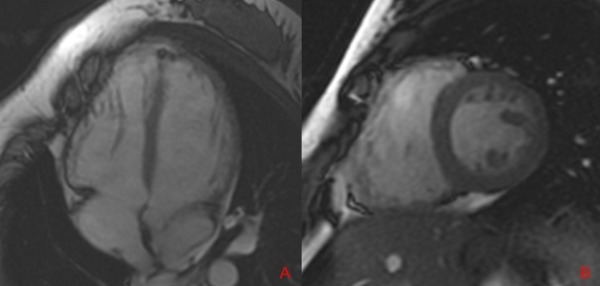
Cardiac MRI demonstrating trabeculations and deep recesses in the right and left ventricle in the four-chamber horizontal long axis view (A) and two-chamber short axis view (B).

Six months later, she had recovered completely from her neurological event. Frequent PVCs continued, and EF was 40-45%. MRI demonstrated a dilated LV and prominent trabeculations along the free wall (Figure 3). The RV showed fibrosis and moderate dilatation without features of arrhythmogenic right ventricular cardiomyopathy. Repeat EP study was done to again risk assess for prophylactic AICD. No VT was inducible. The patient has done well on follow-up at 18 months on warfarin anticoagulation. We present a patient with severe LAA dysfunction in the absence of AF likely related to frequent PVCs and associated with an otherwise unexplained stroke.

## Discussion

We present a young patient with frequent ventricular arrhythmia and severe LAA dysfunction with cardioembolic stroke. The finding of LAA dysfunction and reduced emptying velocity with extraystolic beats has not been described. While LAA dysfunction and the presence of AF is a recognized risk factor for stroke, our patient had no evidence of atrial arrhythmia despite multiple monitors and evaluations.

Our patient had an ablation procedure one year prior to the stroke and thromboembolism from radiofrequency ablation is well described and occurs in approximately 1.3% of patients [3]. However, stroke in this setting typically occurs in the peri-ablation period and would be exceedingly unusual one year post procedure. Further, there was no evidence of thrombus in the LV.

Our patient was diagnosed with prominent myocardial trabeculations which may have been a variant of LVNC cardiomyopathy, and in the setting of ventricular dysfunction, is also a known cause of intramyocardial recess, thrombus, and stroke risk. Estimates for thromboembolic events in LVNC cardiomyopathy range from 10-37% [4]. LVNC, however, was apparent rather late in the course of our patient care and was not apparent at the time of the index event. Further, despite TEE and MRI, there was no evidence of thrombus in the LV. While we cannot conclusively rule out a ventricular cause for micro-thrombus and stroke cause, the finding of LAA dysfunction in this clinical concept, to our knowledge, is a novel finding and suggests at least an additional potential substrate for her cardioembolic presentation.

The LAA emptying velocities decreased by more than 50% during ectopic beats in this patient. LAA dysfunction as an association for stroke is well established even when active thrombus is not found in the LAA. The best established association is with AF, and the LAA is considered a major source of thromboembolism. LAA flow decreases significantly in the same patients during AF compared to sinus rhythm [7]. LAA dysfunction and thrombus formation is very unusual in the absence of AF, even with severely depressed LV function [8-10]. Age, alone, is also an independent association with a negative decline in LAA contraction but has been documented in patients 45 years or older [11].

Our patient was 18 years of age with no evidence of AF and documented specific decrease in LAA function during frequent ventricular ectopy. Ventricular-based pacing (VVI) and associated atrioventricular dyssynchrony has been documented. Dyssynchronous atrial contraction results in significantly larger LAA and atrial electrical remodeling . Alizadeh et al. demonstrated LAA flow velocity decrease during VVI pacing in sinus rhythm compared to those who had AV sequential pacemakers and EF >30% [2]. Several clinical trials have shown increased risk of embolic CVA in patients with dyssynchronous VVI pacing compared to "physiologic" pacing . In our patient, a majority of her ventricular depolarizations were PVCs that, in effect, mimicked dyssynchronous ventricular pacing at times for extended durations, and may have contributed to the morphological, electrical, and functional abnormalities noted in the LAA akin to VVI pacing. It should be noted, however, that AF itself is increased in this setting of dyssynchronous ventricular activation [13] with a risk of development of AF increased at an odds ratio of 2.89 when frequent premature complexes (atrial and ventricular) on electrocardigraphic recordings were noted [14].

Agarwal et al. have demonstrated an association between PVCs and incidental stroke and found that even in the absence of other known risk factors for stroke, PVCs were associated independently with increased incident stroke (hazard ratio 2.09 [95% CI: 1.21-3.58]), and the association was strongest with embolic stroke [15]. LAA dysfunction may be the connecting link between the previously observed association of PVCs and stroke. Although reversal of cardiomyopathy with therapy for PVCs is well established [1], it is unknown whether appendage dysfunction will improve. Other studies have shown that depressed LAA function in patients with mitral stenosis does not completely resolve with valvotomy [16]. Age alone is an independent association with a negative decline in contraction but has been documented in patients 45 years or older. It should be noted that in patients with atrioventricular dyssynchrony, as occurs with frequent PVCs, left atrial pressures are elevated with subsequent decline with normal ventricular systole. In our patient, the LAA velocities were consistently depressed with both PVCs as well as normal sinus beats ranging from 30-50 cm/second measured velocities (normal value 50-64 cm/second). However, on average, lower velocities were noted during the PVCs. It is unknown whether the PVCs themselves create LAA dysfunction or an intermediate pathogenic step involves relatively persistently elevated left atrial pressures. Regardless, however, the potential for enhanced stroke risk likely exists in this situation [17].

Thus, to our knowledge, this is the first report of documented PVC-related LAA dysfunction in a patient with an embolic cerebrovascular event. While other potential causes including LVNC and prior ablation cannot be conclusively excluded, further study is required to assess the frequency of LAA dysfunction and stroke risk in patients with frequent PVCs causing atrioventricular dyssynchronous activation. Although the patient's prominent trabeculations was the most likely source of the PVCs, the question remains whether or not the PVCs resulted in LAA dysfunction which in turn precipitated her embolic stroke. Although we cannot delineate the exact cause of the stroke, it is interesting to consider this possible relationship of LAA dysfunction and frequent PVCs. Further studies are needed to elucidate this phenomenon and clarify the connection between these two seemingly separate entities.
